# Supplementary material for: EnzML: multi-label prediction of enzyme classes using InterPro signatures
Source: BMC Bioinformatics. 2012 Apr 25;13:61. doi: 10.1186/1471-2105-13-61 (PMC3483700; doi:10.1186/1471-2105-13-61)
Supplement: Addtional file 5 — The Java code to format the data files, evaluate and predict. The file enzml_java_code.tar.gz contains the Java code used to format database data to ARFF and XML formats, to execute cross and train-test (jackknife) evaluations and to record evaluation results to database. More information is included in the readme.txt file and the Javadoc files. The code can be used with a MySQL database. To use a different database software, other JDBC drivers might be required. [file 1471-2105-13-61-S5.gz › java_code/enzml2011/doc/test/mulan/learn/traintest/EvaluationMetricsTest.html]

EvaluationMetricsTest


---


|  |  |  |
| --- | --- | --- |
| **Overview** \| **Package** \| **Class** \| **Use** \| **Tree** \| **Deprecated** \| **Index** \| **Help** | |  |
| PREV CLASS   **NEXT CLASS** | **FRAMES**    **NO FRAMES**     **All Classes** |  |
| SUMMARY: NESTED \| FIELD \| CONSTR \| METHOD | DETAIL: FIELD \| CONSTR \| METHOD |  |


---


## test.mulan.learn.traintest Class EvaluationMetricsTest

```
java.lang.Object
  junit.framework.Assert
      junit.framework.TestCase
          test.mulan.learn.traintest.EvaluationMetricsTest
```

**All Implemented Interfaces:**: junit.framework.Test

---

``` public class EvaluationMetricsTest extends junit.framework.TestCase ```

Tests the evaluation metrics results: accuracy, precision, recall etc.

**Version:**
:   2 May 2011

**Author:**
:   Luna De Ferrari luna.deferrari-at-ed.ac.uk

---

**Constructor Summary**

`EvaluationMetricsTest()`


| **Method Summary** | |
| --- | --- |
| `static void` | `basicPredictionsTest(TrainTestExperimenter exp, java.lang.String expectedPredictions)` |
| `static uk.ac.ed.inf.utils.database.TableRow` | `basicResultsTest(TrainTestExperimenter experimenter)` |
| `static int[]` | `getFilteredArffs(int trainArffId, int oldArff)` |
| `static int` | `getInstance1_2_empty_ArffId()` |
| `static int` | `getInstance1_2ArffId()` |
| `static int` | `getInstance3ArffId()` |
| `void` | `testEvalResultsInstance12()`             Train on instances 1 and 2 and test on instances 1 and 2: should have perfect precision/accuracy/recall |
| `void` | `testEvalResultsTrainInst12TestInst3()`             Train on instances 1 and 2 and test on instances 3: should have zero precision/accuracy/recall |
| `static void` | `testMetrics_Train_1_2_empty_Test_3(uk.ac.ed.inf.utils.database.TableRow row)` |
| `static void` | `testMetrics_Train_1_2_Test_3(uk.ac.ed.inf.utils.database.TableRow row)` |
| `static void` | `testMetricsTrain_1_2_Test_1_2(uk.ac.ed.inf.utils.database.TableRow row)` |
| `void` | `testTrain_1_2_empty_Test_3()`             Train on instances 1 and 2 and empty and test on instances 3, which is empty once filtered |

**Methods inherited from class junit.framework.TestCase**

`countTestCases, getName, run, run, runBare, setName, toString`

**Methods inherited from class junit.framework.Assert**

`assertEquals, assertEquals, assertEquals, assertEquals, assertEquals, assertEquals, assertEquals, assertEquals, assertEquals, assertEquals, assertEquals, assertEquals, assertEquals, assertEquals, assertEquals, assertEquals, assertEquals, assertEquals, assertEquals, assertEquals, assertFalse, assertFalse, assertNotNull, assertNotNull, assertNotSame, assertNotSame, assertNull, assertNull, assertSame, assertSame, assertTrue, assertTrue, fail, fail, failNotEquals, failNotSame, failSame, format`

**Methods inherited from class java.lang.Object**

`equals, getClass, hashCode, notify, notifyAll, wait, wait, wait`

**Constructor Detail**

### EvaluationMetricsTest

```
public EvaluationMetricsTest()
```


**Method Detail**

### testEvalResultsInstance12

```
public void testEvalResultsInstance12()
```

:   Train on instances 1 and 2 and test on instances 1 and 2: should have
    perfect precision/accuracy/recall

---


### testEvalResultsTrainInst12TestInst3

```
public void testEvalResultsTrainInst12TestInst3()
```

:   Train on instances 1 and 2 and test on instances 3: should have zero
    precision/accuracy/recall

---


### testTrain\_1\_2\_empty\_Test\_3

```
public void testTrain_1_2_empty_Test_3()
```

:   Train on instances 1 and 2 and empty and test on instances 3, which is
    empty once filtered

---


### basicPredictionsTest

```
public static void basicPredictionsTest(TrainTestExperimenter exp,
                                        java.lang.String expectedPredictions)
```

---


### basicResultsTest

```
public static uk.ac.ed.inf.utils.database.TableRow basicResultsTest(TrainTestExperimenter experimenter)
```

---


### getFilteredArffs

```
public static int[] getFilteredArffs(int trainArffId,
                                     int oldArff)
```

---


### getInstance1\_2\_empty\_ArffId

```
public static int getInstance1_2_empty_ArffId()
```

---


### getInstance1\_2ArffId

```
public static int getInstance1_2ArffId()
```

---


### getInstance3ArffId

```
public static int getInstance3ArffId()
```

---


### testMetrics\_Train\_1\_2\_empty\_Test\_3

```
public static void testMetrics_Train_1_2_empty_Test_3(uk.ac.ed.inf.utils.database.TableRow row)
```

---


### testMetrics\_Train\_1\_2\_Test\_3

```
public static void testMetrics_Train_1_2_Test_3(uk.ac.ed.inf.utils.database.TableRow row)
```

---


### testMetricsTrain\_1\_2\_Test\_1\_2

```
public static void testMetricsTrain_1_2_Test_1_2(uk.ac.ed.inf.utils.database.TableRow row)
```


---


|  |  |  |
| --- | --- | --- |
| **Overview** \| **Package** \| **Class** \| **Use** \| **Tree** \| **Deprecated** \| **Index** \| **Help** | |  |
| PREV CLASS   **NEXT CLASS** | **FRAMES**    **NO FRAMES**     **All Classes** |  |
| SUMMARY: NESTED \| FIELD \| CONSTR \| METHOD | DETAIL: FIELD \| CONSTR \| METHOD |  |


---
